# Epigenetic Signatures of Ageing in Asian Elephants Revealed by Reduced Representation Bisulphite Sequencing

**DOI:** 10.1111/eva.70236

**Published:** 2026-04-13

**Authors:** Kana Arai, Miho Inoue‐Murayama

**Affiliations:** ^1^ Wildlife Research Center, Kyoto University Kyoto Japan; ^2^ Graduate School of Science, Kyoto University Kyoto Japan

**Keywords:** ageing, Asian elephants, DNA methylation, epigenetics, genome‐wide, reduced representation bisulphite sequencing

## Abstract

Accurate age estimation is essential for understanding life‐history variation, modelling population dynamics and informing conservation strategies, yet remains challenging for long‐lived species. Here, we developed a genome‐wide DNA methylation‐based epigenetic clock for Asian elephants ( *Elephas maximus* ), an endangered species, using reduced representation bisulphite sequencing (RRBS). Genome‐wide methylation profiles from 91 blood samples yielded 144,611 candidate CpG sites, of which 389 CpG sites were strongly associated with chronological age. The final model predicted age with high accuracy (*r* = 0.96, MAE = 4.82 years), corresponding to a relative error of 6.06%, comparable to epigenetic clocks developed for humans and other non‐model species. Longitudinal analyses revealed heterogeneous epigenetic ageing trajectories, with most individuals showing increases in epigenetic age over time, while others exhibited relatively slower or minimal change, indicating a potential inter‐individual variation in ageing processes. Functional enrichment analyses revealed that age‐associated CpGs were enriched in genes and pathways related to development, neurogenesis, metabolism and social or physiological regulation, including oxytocin, apelin and melanogenesis signalling. Age‐related methylation changes were characterised by predominant hypermethylation in CpG islands and genic regions, consistent with patterns reported across mammals. Together, these findings demonstrate that epigenetic clocks capture biologically meaningful features of ageing that could be linked to life‐history traits. As long‐lived mammals with complex social systems and extended reproductive capacity, Asian elephants provide a valuable comparative model for investigating the molecular architecture of ageing. This study contributes to the growing field of comparative epigenetic ageing and highlights the potential of DNA methylation‐based approaches to inform evolutionary and conservation‐relevant research when applied in appropriate ecological and methodological contexts.

## 1 Introduction

Age is a fundamental biological parameter that influences an organism's physiology, reproductive potential and survival. At the individual level, it shapes performance by affecting the ability to compete for mates and resources, allocate investment to reproduction and maintain somatic health (Kirkwood and Austad 2000; Nussey et al. 2013). These age‐related changes underpin patterns of ageing and senescence, which are key life‐history traits shaped by natural selection and reflect trade‐offs between growth, reproduction and survival across species (Lemaître et al. 2020). At the population level, age structure is a critical determinant of survival and mortality patterns, making it central to understanding population dynamics and viability, particularly in wild populations lacking long‐term individual‐based monitoring (Gaillard and Lemaître 2017; Heydenrych et al. 2021). Consequently, accurate age information is essential for informing in situ conservation strategies, including demographic inference and the evaluation of management interventions (Healy et al. 2019; Jones et al. 2014). In contrast, age is often well documented in ex situ populations, where it underpins veterinary care, dietary planning, reproductive management and behavioural enrichment across life stages (Hecht 2021; Oh et al. 2025). These populations also provide valuable opportunities for ageing research, as individuals can be monitored longitudinally under relatively controlled conditions with detailed health, reproductive and behavioural records, enabling insights into age‐related biological processes that are difficult to obtain in wild systems.

Despite its importance, accurately estimating age remains challenging for many animal species, particularly those lacking known birth records or clear external indicators of ageing. In some taxa, age can be inferred from physical structures, such as otoliths and scales in fish (Gunn et al. 2008), skeletochronology in sea turtles (Tomaszewicz et al. 2015), tooth cementum layers in pinnipeds and terrestrial mammals (Scheffer 1950; Goodwin and Ballard 1985; Gable et al. 2023), or skin speckling patterns in dolphins (Krzyszczyk and Mann 2012). However, these approaches are often constrained by ecological and methodological limitations, including the need for capture, close handling or post‐mortem samples. In addition, many traits used for age estimation are influenced by environmental or behavioural factors, which can confound interpretation. For example, tooth wear reflects both age and feeding behaviour (Hohn and Fernandez 1999), while assessments of skin speckling are subjective and depend on complete visual access to the animal (Krzyszczyk and Mann 2012). Long‐term behavioural studies can provide reliable age information, but they typically require decades of continuous monitoring to capture full lifespans. Other proxy‐based approaches, such as body length (Veylit et al. 2021), plumage variation in birds (Weimerskirch et al. 1989) or larval morphology in molluscs (Ernande et al. 2003), generally yield only coarse estimates of life stage, particularly once growth plateaus in adulthood. Similarly, although mark–recapture methods are widely used to estimate survival and population dynamics (Reinke et al. 2019; Wilkinson and Brunet‐Rossinni 2009), precise age and individual growth trajectories often remain difficult to determine.

In Asian elephants ( *Elephas maximus* ), despite ongoing efforts, age estimation remains challenging, with many current approaches being subjective and difficult to standardise. Traditional methods rely on long‐term observational records, assessments of deceased individuals or evaluations of external morphological traits, including shoulder height, tusk size, ear shape, foot dimensions (Arivazhagan and Sukumar 2008; Fernando et al. 2022) and dung bolus circumference (Mohanarangan et al. 2022). While these approaches are non‐invasive and practical under field conditions—and can perform reasonably well for younger individuals such as calves and subadults—their reliability declines in adults, where growth slows and individual variation increases (Arivazhagan and Sukumar 2008). More precise estimates can be obtained from tooth eruption and wear patterns based on cranio‐dental features, however, these methods typically require immobilisation of live individuals or post‐mortem examination and are often derived from limited sample sizes, restricting their broader applicability (Rasmussen et al. 2008; Roth and Shoshani 1988). As a result, external indicators become increasingly indistinct with age, making objective and scalable age estimation particularly difficult for mature individuals. Such uncertainty can bias demographic analyses and hinder accurate assessments of survival, reproduction and population viability.

Advances in molecular ageing studies have provided new tools for quantifying age‐related biological change. DNA methylation‐based biomarkers estimate chronological and epigenetic age by measuring age‐associated changes at specific CpG sites across the genome (Horvath 2013; Horvath and Raj 2018; Jarman et al. 2015). DNA methylation is one of the most well‐characterised epigenetic modifications, playing a key role in gene regulation, chromatin organisation, genomic stability and ageing, without altering the underlying DNA sequence (Moore et al. 2013). Technological advances in sequencing have enabled the quantification of methylation levels at specific CpG sites, facilitating the development of accurate ‘epigenetic clocks’. From an evolutionary perspective, DNA methylation patterns associated with ageing may reflect conserved and lineage‐specific regulatory strategies through which species modulate longevity, stress resistance and late‐life performance (Mc Auley 2023). Initially developed in humans (Horvath 2013), epigenetic clocks have since been extended to a growing range of species, including mice ( *Mus musculus* ) (Petkovich et al. 2017; Stubbs et al. 2017), naked mole rats ( *Heterocephalus glaber* ) (Horvath et al. 2022; Kerepesi et al. 2022), felids (Qi et al. 2024), chimpanzees ( *Pan troglodytes* ) (Ito et al. 2018), bats ( *Myotis bechsteinii* ) (Wright et al. 2018; Wilkinson et al. 2021) and cetaceans (Beal et al. 2019; Polanowski et al. 2014; Bors et al. 2021). Beyond their applied utility for age estimation, these clocks have become powerful tools in comparative biology, enabling investigations of how ageing trajectories and molecular mechanisms vary across species with divergent life‐histories and lifespans (Klughammer et al. 2023; Lu et al. 2023). Comparative epigenetic studies have revealed that long‐lived species often exhibit different rates of epigenetic drift or altered age‐associated methylation dynamics, consistent with evolutionary investment in enhanced somatic maintenance and genomic stability (Bertucci‐Richter and Parrott 2023). Together, these patterns offer insights into the evolutionary mechanisms underlying longevity and trade‐offs between growth, reproduction and somatic maintenance, and highlight the value of extending genome‐wide epigenetic ageing analyses to long‐lived, non‐model species.

Epigenetic clocks can be broadly categorised into different generations based on their design and objectives. First‐generation clocks, such as Horvath's multi‐tissue clock and Hannum's blood‐based clock, are trained to predict chronological age using CpG sites with consistent age‐associated methylation patterns (Horvath 2013; Hannum et al. 2013). In contrast, second‐ and third‐generation epigenetic clocks incorporate additional clinical biomarkers, physiological measures or health‐related outcomes, and are designed to capture inter‐individual differences in mortality risk, health‐related phenotypes and rates of multi‐organ system decline, rather than chronological age alone (Levine et al. 2018; Lu et al. 2019; Belsky et al. 2022). While these later‐generation clocks have provided valuable insights into humans (Oblak et al. 2021), their application in non‐human species remains limited. Developing such approaches in elephants could offer valuable insights into ageing, health and life‐history variation. Accordingly, the present study focuses on constructing a DNA methylation‐based age estimation model aligned with first‐generation epigenetic clocks, providing a foundation for future development of more advanced biomarkers of biological ageing.

Asian elephants are endangered, with populations increasingly threatened by habitat loss, human‐elephant conflict and poaching (Williams et al. 2020). Effective conservation, therefore, depends on robust demographic data and accurate assessments of population viability. While DNA methylation‐based age estimation offers an objective molecular framework that can overcome limitations of traditional age estimation methods, its application to free‐ranging elephant populations remains constrained by the practical difficulty of obtaining high‐quality biological samples. In particular, blood sampling typically requires immobilisation or relies on opportunistic veterinary procedures. These challenges highlight the importance of evaluating both the potential and practical limitations of epigenetic age estimation in conservation contexts. Accordingly, epigenetic approaches should be viewed as complementary tools, whose applicability depends on sampling feasibility, study design and integration with existing ecological and demographic data. In contrast, captive elephant populations provide a valuable system for ageing research, as known or well‐constrained ages, repeated sampling opportunities and detailed longitudinal records enable investigation of age‐associated molecular changes and inter‐individual variation that are difficult to assess in wild populations. Epigenetic age estimates may also help refine or validate incomplete records, supporting long‐term management of captive populations (Clubb and Mason 2002).

Asian elephants are socially complex, large‐brained mammals (de Silva et al. 2011; Shoshani et al. 2006) with exceptional longevity, with some individuals living into their 80s (Lahdenperä et al. 2014). They reach sexual maturity in their early teens and can reproduce into old age, with no clear period of reproductive senescence (Lahdenperä et al. 2014). Despite their large body size and extended lifespan, elephants experience a remarkably low incidence of age‐related diseases such as cancer (Abegglen et al. 2015), suggesting the presence of evolved mechanisms of somatic maintenance and physiological adaptations that mitigate ageing‐associated decline and promote disease resistance. Their advanced cognition and strong social bonds further parallel aspects of human ageing, making elephants a compelling comparative model for studying ageing in a long‐lived, non‐model species (Chusyd et al. 2021). Investigating age‐associated DNA methylation in Asian elephants, therefore, provides a unique opportunity to explore how longevity, reproductive strategies and disease resistance are shaped by evolutionary adaptations in the ageing process (Crofts et al. 2024).

Previous epigenetic age estimation studies in elephants have employed either the MammalMethylChip40 array or targeted RT‐PCR‐based approaches (Arai et al. 2023; Prado et al. 2021). While array‐based methods target highly conserved CpG sites across mammals, they are constrained by predefined probe sets, and targeted approaches, although cost‐efficient, provide limited genomic resolution. In contrast, reduced representation bisulphite sequencing (RRBS) enables genome‐wide profiling of DNA methylation at single‐CpG resolution, allowing the identification of novel age‐associated CpG sites without prior probe design or assumptions from other species. By enriching CpG‐dense regions, RRBS achieves a balance between genomic coverage and sequencing depth (Gu et al. 2011; Klughammer et al. 2015). Although successfully applied in other long‐lived and non‐model species, including naked mole rats (Kerepesi et al. 2022) and baboons ( *Papio cynocephalus* ) (Anderson et al. 2021), it has not yet been applied to Asian elephants.

Here, we use RRBS to generate high‐resolution, genome‐wide DNA methylation profiles in Asian elephants and identify CpG sites strongly associated with age. We then integrate these data with machine‐learning approaches to develop a species‐specific epigenetic clock, leveraging recently available genomic resources for this species. Beyond age estimation, this work contributes to the comparative biology of ageing by characterising molecular ageing patterns in one of the longest‐lived terrestrial mammals. Together, this study demonstrates how molecular ageing tools could inform conservation‐relevant questions while advancing evolutionary perspectives on ageing, providing a foundation for future studies on the interactions among life history, health and environmental influences on ageing trajectories.

## 2 Materials and Methods

### 2.1 Study Animal and Sample Collection

There are 31 zoos in Japan housing a total of 81 captive Asian elephants (22 males and 59 females). Most zoos in Japan conduct husbandry training to facilitate blood collection during routine veterinary health examinations. However, not all individuals are trained, which makes it difficult to collect samples from every elephant, particularly males during the musth period. Even well‐trained individuals may unexpectedly resist health examinations. To minimise stress and avoid invasive sampling as much as possible, our study focused on 30 well‐trained individuals (3 males and 27 females) who were cooperative during sampling.

A total of 91 blood samples were collected across 12 zoos in Japan between 2004 and 2023 during veterinary health examinations, providing a longitudinal dataset (Table 1). Whole blood samples from either an ear or leg vein were collected directly into an EDTA tube during regular veterinary examinations. Known birthdates or estimated birth years were obtained from studbook records provided by each zoo staff. Individuals had known birthdates if they were born in captivity in Japan or in a range country (Myanmar, Laos or Thailand). Those with estimated birth years were wild‐born individuals, with estimates likely determined by brokers upon importation or by zoo staff based on morphometric measurements. Individuals with estimated birth years were born before the late 1970s, reflecting the historical management of captive populations, as captive breeding was not widely implemented as a population management tool until the 1980s. Although uncertainty in chronological age can influence epigenetic age model performance, its impact in this study is likely limited as most individuals had known birthdates, with only a small subset relying on estimated birth years (Data S1). Therefore, any residual uncertainty is expected to be small relative to the overall lifespan of elephants. All samples were stored in a −80°C or −20°C freezer until DNA extraction.

**TABLE 1:** Distribution of Asian elephant samples across age classes and sex used for the analyses.

Age class	Age range (years)	Female (*n*)	Male (*n*)	Total (*n*)
Calves	< 1	3	4	7
Juveniles	1–5	—	4	4
Subadults	5–20	30	11	41
Adults	20–50	30	—	30
Seniors	> 50	9	—	9
Total		72	19	91

### 2.2 DNA Extraction

DNA was extracted using the DNeasy Blood & Tissue Kit (QIAGEN, GmbH, Hilden, Germany) following the manufacturer's protocol. DNA yield was measured using a Qubit 4 Fluorometer with the Qubit dsDNA Assay Kit (Thermo Fisher Scientific, San Jose, CA, USA), with 1 μL of input DNA. The extracted DNA was stored at −20°C until further use.

### 2.3 DNA Methylation Data Using RRBS

RRBS libraries were prepared using the Zymo‐Seq RRBS Library Kit (Zymo Research, Irvine, CA, USA) with ~300 ng of DNA per sample, following the manufacturer's protocol. The libraries were validated and quantified using an Agilent 2200 TapeStation (Agilent Technologies) to assess concentration and fragment size distribution. Libraries were pooled and sequenced on an Illumina NovaSeq X Plus at Macrogen Japan, generating 150 bp paired‐end reads using a 10B flow cell. To compensate for the low complexity of the libraries, a 20% PhiX control was spiked in.

The quality of sequenced reads was assessed using *FastQC* (version 0.12.1; Andrews 2010). Reads were trimmed and quality‐filtered using *Trim Galore!* (version 0.6.10; Krueger 2012) to remove adapters and low‐quality sequences (Phred score < 20), with the *‐rrbs* option applied. Trimmed reads were aligned to a bisulphite‐converted index of the Asian elephant genome from NCBI (GCF_024166365.1) using *Bismark* (version 0.24.2; Krueger and Andrew 2011) with *Bowtie2* (Langmead and Salzberg 2012). Methylation calling was performed using Bismark's methylation extractor with default settings, where site‐specific DNA methylation levels were estimated for each sample and CpG site by calculating the proportion of methylated reads, as the extractor provides both the methylated and total read counts. Following Anderson et al. (2021), CpG sites were filtered to retain those with a mean coverage depth of at least 5× and mean methylation levels between 0.1 and 0.9, excluding constitutively hyper‐ or hypomethylated sites. CpG sites with missing data in ≥ 5% of individuals were excluded. After filtering, 144,611 CpG sites were retained for downstream analysis. Any remaining missing values were imputed using a k‐nearest neighbours approach implemented in the R package *impute*, with default parameters (Hastie et al. 2001).

### 2.4 Development of the Epigenetic Clock

#### 2.4.1 Data Splitting and Pre‐Processing

A methylation profile table (feature table) containing CpG sites with sufficient coverage across all samples was used for model development. Samples were randomly assigned to either the training dataset (65 samples), which was used to train the model by fitting its parameters, or the testing dataset (26 samples), which was used to evaluate the model's performance. The split was performed using the *createDataPartition* function in the *caret* R package, which applies stratified random sampling to maintain a balanced distribution of age across the training and testing datasets (Kuhn 2008). Following Anderson et al. (2021), methylation ratios (proportion of methylated counts to total counts at each CpG site) were quantile normalised within each sample to a standard normal distribution using the *qqnorm* function in R.

#### 2.4.2 Model Tuning

A penalised regression model was used, with the predictor variables representing normalised DNA methylation levels at 144,611 candidate clock CpG sites across the genome, and the response variable being chronological age. Specifically, the *glmnet* R package (version 4.1‐8) was used to implement an elastic net regression model (Friedman et al. 2010). Elastic net regression is well‐suited for high‐dimensional datasets with more features than observations, such as DNA methylation data, where the number of CpG sites exceeds the number of samples. By combining the regularisation properties of ridge regression (L2 penalty), which shrinks coefficients continuously, and lasso regression (L1 penalty), which can set some coefficients to zero, this approach balances feature selection and coefficient shrinkage, improving model interpretability and reducing overfitting (Friedman et al. 2010). To identify the optimal balance between ridge and lasso penalties, a grid search was conducted across alpha values ranging from 0 (ridge regression) to 1 (lasso regression) in increments of 0.01. For each alpha value, the regularisation lambda was selected as the value minimising mean squared error through internal *n*‐fold cross‐validation within the training dataset. The optimal alpha was selected based on the highest cross‐validated *R* ^2^ between predicted and true chronological age within the training dataset.

Additionally, model tuning involved feature pre‐selection followed by regression to assess performance improvements. Feature selection was carried out in two steps: CpG sites were first filtered using Pearson's correlations (*r*) > 0.5 with age, followed by penalised regression or support vector machine regression with a radial basis function (SVMr), implemented using the *e1071* R package (version 1.7‐16; Meyer et al. 2024), following Qi et al. (2024).

#### 2.4.3 Model Evaluation and Assembling Predictions

As 21 individuals were sampled repeatedly over time, model evaluation and parameter tuning were performed using leave‐one‐individual‐out cross‐validation (LOIOCV) during feature selection and regression analysis to validate all models. LOIOCV accounts for individuals sampled multiple times by ensuring that data from one individual is excluded for validation at each iteration to prevent overfitting. This process is repeated as many times as the number of individuals, with the regression model trained on the remaining samples and then used to predict the age of the held‐out sample. By maximising the size of the training set in each iteration, this process enhances model robustness and helps assess whether the optimised model is prone to overfitting.

Final model performance was evaluated using the test dataset to assess its generalisability and precision. Accuracy was assessed using metrics including mean absolute error (MAE) and coefficient of determination (*R* ^2^) between predicted age (DNAm age) and chronological age to indicate how well CpG methylation patterns explain variability in age.

### 2.5 Exploration of Biological Variation in DNA Methylation

#### 2.5.1 Methylation‐Based Longitudinal Age Predictions

Longitudinal samples were available for 21 individuals, allowing limited within‐individual analyses of changes in DNAm age over time. To maximise temporal separation, only the first and last sampling timepoints were retained for each individual. Changes in DNAm age were assessed relative to the elapsed chronological time between these two sampling points to assess whether DNAm age increased in accordance with chronological ageing. To minimise the influence of negligible temporal separation on slope estimation, individuals were excluded if the time interval between their first and last samples was less than a day. This criterion removed same‐day or near‐duplicate sampling events that are unlikely to provide meaningful information on longitudinal epigenetic change. All remaining individuals had an interval which spanned from approximately 1 to 14 years between samples and were retained for longitudinal slope estimation (Table 2).

**TABLE 2:** Chronological age at first and last sampling and resulting longitudinal sampling interval for individuals with repeated blood samples (*n* = 17).

IndID	Sex	Chronological age at first sampling (years)	Chronological age at last sampling (years)	Interval (years)
10	F	6.46	7.93	1.47
11	F	8.95	10.48	1.53
12	F	10.78	12.24	1.46
13	M	17.64	19.19	1.55
14	F	20.22	21.75	1.53
15	F	30.34	31.78	4.46
19	M	0.25	3.64	3.39
20	F	7.90	13.04	5.14
21	M	6.59	12.00	5.41
22	F	6.46	12.96	6.50
23	F	10.20	15.79	5.59
24	F	16.39	19.27	2.78
25	F	28.25	41.67	13.42
26	F	29.06	42.48	13.22
27	F	31.75	43.90	12.15
28	F	38.35	52.95	14.60
29	F	32.47	46.38	13.91

*Note:* Intervals represent the time elapsed between the first and last samples (excluding those with time intervals of < 1 day), used for within‐individual analyses of changes in DNA methylation‐based age.

#### 2.5.2 Age Group DNA Methylation Differences and Performance

Continuous age was retained for all regression‐based analyses and epigenetic clock development, with categorical age used to facilitate life‐history‐based interpretation of methylation patterns. Here, age was categorised into five biologically defined groups reflecting major life‐history stages of Asian elephants: calves (< 1 year), juveniles (1–5 years), subadults (5–20 years), adults (20–50 years) and seniors (> 50 years). These boundaries were defined based on the knowledge of elephant growth, development and reproductive biology. Calves (< 1 year) represent the dependent neonatal stage characterised by rapid growth and maternal reliance (Nair 1989; Revathe et al. 2020), while juveniles (1–5 years) reflect early post‐weaning development with continued growth (Sukumar 1989). The subadult category (5–20 years) captures the prolonged period of growth and delayed sexual maturation, before full reproductive activity (de Silva et al. 2013; Hayward et al. 2014; Sukumar 1989). The transition at 20 years between subadults and adults was selected based on cessation of growth and average age of first reproduction and peak fertility in females (Lahdenperä et al. 2014; Hayward et al. 2014). Individuals older than 50 years were classified as senior, reflecting late‐life stages associated with declining survival probabilities and age‐related physiological changes (de Silva et al. 2013). These categories were used for exploratory and comparative analyses, allowing visualisation and testing of potential stage‐specific differences in DNA methylation patterns that may not be linear across the lifespan. Group‐wise differences in DNAm age were assessed using a one‐way analysis of variance (ANOVA) followed by post hoc pairwise comparisons. To further explore age‐associated methylation patterns, principal component analysis (PCA) was performed using the *FactoMineR* R package (Lê et al. 2008), with scaled data, focusing on age‐associated CpG sites.

#### 2.5.3 Sex Associated DNA Methylation

To test for sex differences at individual CpG sites included in the epigenetic age estimation model, a site‐wise multiple linear regression was applied. Methylation of each of the 389 CpG sites was modelled as a function of predicted age, sex and their interaction. *p*‐values were adjusted using the Benjamini‐Hochberg FDR method (FDR < 0.05). To assess sex differences in epigenetic age acceleration (Δage), residuals from a linear model of DNAm age regressed on chronological age (Δage residuals) were analysed using a generalised linear model with sex and age as predictors. PCA was also performed to further explore associations between DNA methylation profiles and sex.

### 2.6 Epigenetic Clock Enrichment Analysis

Genomic locations and nearest genes of age‐related CpG sites were annotated using the Asian elephant genome (GCF_024166365.1) via NCBI Genome Data Viewer. Genomic contexts were classified by CpG density (islands, shores, other) and genomic features (promoters, introns, exons, intergenic regions). Promoter regions were defined as ±2000 bp from transcription start sites of the corresponding gene, and CpG shores as 2000 bp regions flanking either side of a CpG island. To understand the functional relevance of age‐associated CpG methylation in Asian elephants, enrichment analyses were conducted to identify enriched biological pathways and Gene Ontology (GO) terms associated with age‐related CpG sites. These analyses were performed using Enrichr (Chen et al. 2013) and g:Profiler (Raudvere et al. 2019), with the human genome (hg38/GRCh38) used as reference background. Multiple testing correction was applied using FDR (adjusted *p* < 0.05).

## 3 Results

### 3.1 Global Changes in Methylation With Age

On average, 44.9 million reads per sample were aligned to the Asian elephant genome. Following quality control and filtering steps, a total of 144,611 candidate CpG sites were identified across the genome, with a global mean DNA methylation level of 65.9%. This global methylation level did not correlate significantly with chronological age (*r* = 0.05, *p* = 0.65), and remained stable across individuals, consistently showing moderate methylation levels (i.e., more than 0.5 across all samples) (Figure S1a). To further investigate global changes in Asian elephant methylation, Pearson's correlation coefficients between methylation levels and age were calculated at each CpG site. Approximately 12% (16,944 sites) of the total CpGs were significantly correlated with age (*p* < 0.05), and 3774 CpG sites remained significant after FDR correction (Figure S1b).

### 3.2 Epigenetic Clock Calibration and Composition

**FIGURE 1:**
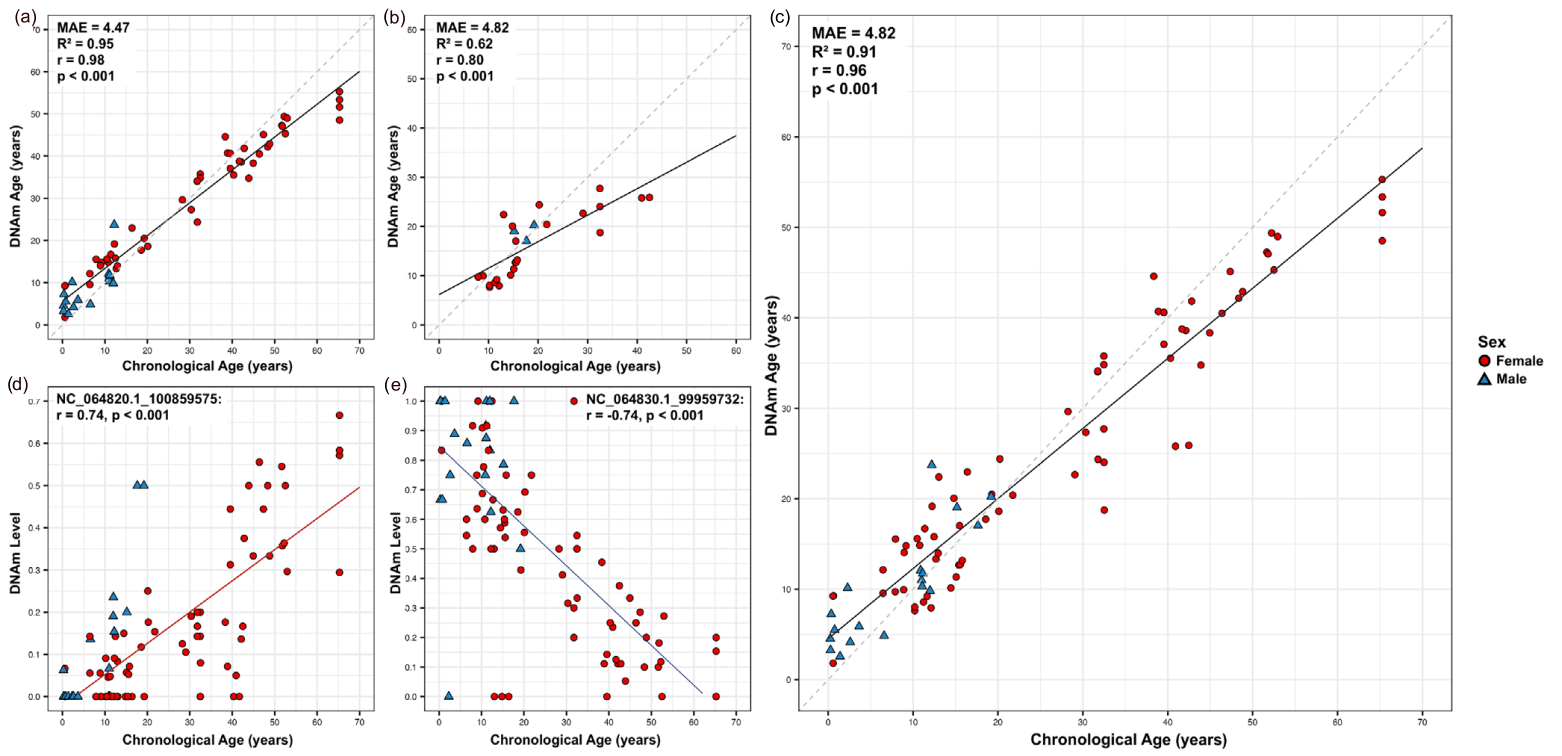
Epigenetic clock estimates for Asian elephants based on genome‐wide DNA methylation profiles generated using reduced representation bisulphite sequencing (RRBS). (a) Performance of the Asian elephant epigenetic clock in the training dataset and (b) testing dataset. (c) Predicted DNA methylation age (DNAm age) from the final epigenetic clock model constructed using elastic net regression with leave‐one‐individual‐out cross‐validation (LOIOCV). Examples of individual CpG sites showing the strongest (d) positive and (e) negative correlations with chronological age. Each point represents a single blood sample (*n* = 91) taken from 30 individuals. Multiple samples from the same individual with longitudinal data were available (*n* = 21; See Data S1). Model performance is indicated by Pearson's correlation coefficient (*r*), coefficient of determination (*R* ^2^) and mean absolute error (MAE). Solid black lines indicate regression lines in panels (a–c); red and blue lines represent methylation trends in (d) and (e), respectively. The grey dashed line represents *y* = *x*. Female samples are shown as red circles, and male samples are represented as blue triangles.

Starting with a dataset of methylation levels from 144,611 CpG sites, the workflow that combined feature selection based on Pearson's correlation (*r* > 0.5), followed by elastic net regression, produced the best‐performing epigenetic clock model (Figure 1; performance metrics for all feature selection and regression methods are summarised in Table S1, Data S1). A total of 389 CpG sites met the correlation threshold and were used to train the elastic net model, incorporating LOIOCV to provide an unbiased estimate of the epigenetic clock (Figure 1a). The model was then tested using the same procedure to evaluate predictive reliability (Figure 1b). Consistent performance between training and testing data, indicated by similar mean absolute errors (MAEs), suggested minimal overfitting. The unified test predictions from the cross‐validation were strongly correlated with chronological age (*r* = 0.96, *p* = 2.2e‐16) and yielded accurate predictions across samples. Our epigenetic clock model achieved a MAE of 4.82 years (Figure 1c), which corresponds to a relative error of 6.06% based on the maximum recorded lifespan of Asian elephants (79.6 years; de Magalhães and Costa 2009).

All 389 CpG sites used in the model were significantly correlated with age, exhibiting distinct patterns of methylation change. Among them, 288 sites showed positive and 101 showed negative correlations with chronological age (Data S2). Eight CpG sites had particularly strong correlations > 0.7, with the most positively correlated site at *r* = 0.74 (*p* = 2.2e‐16) and the most negatively correlated site at *r* = −0.74 (*p* = 2.2e‐16) (Figure 1d,e).

### 3.3 Methylation‐Based Longitudinal Age Predictions

**FIGURE 2:**
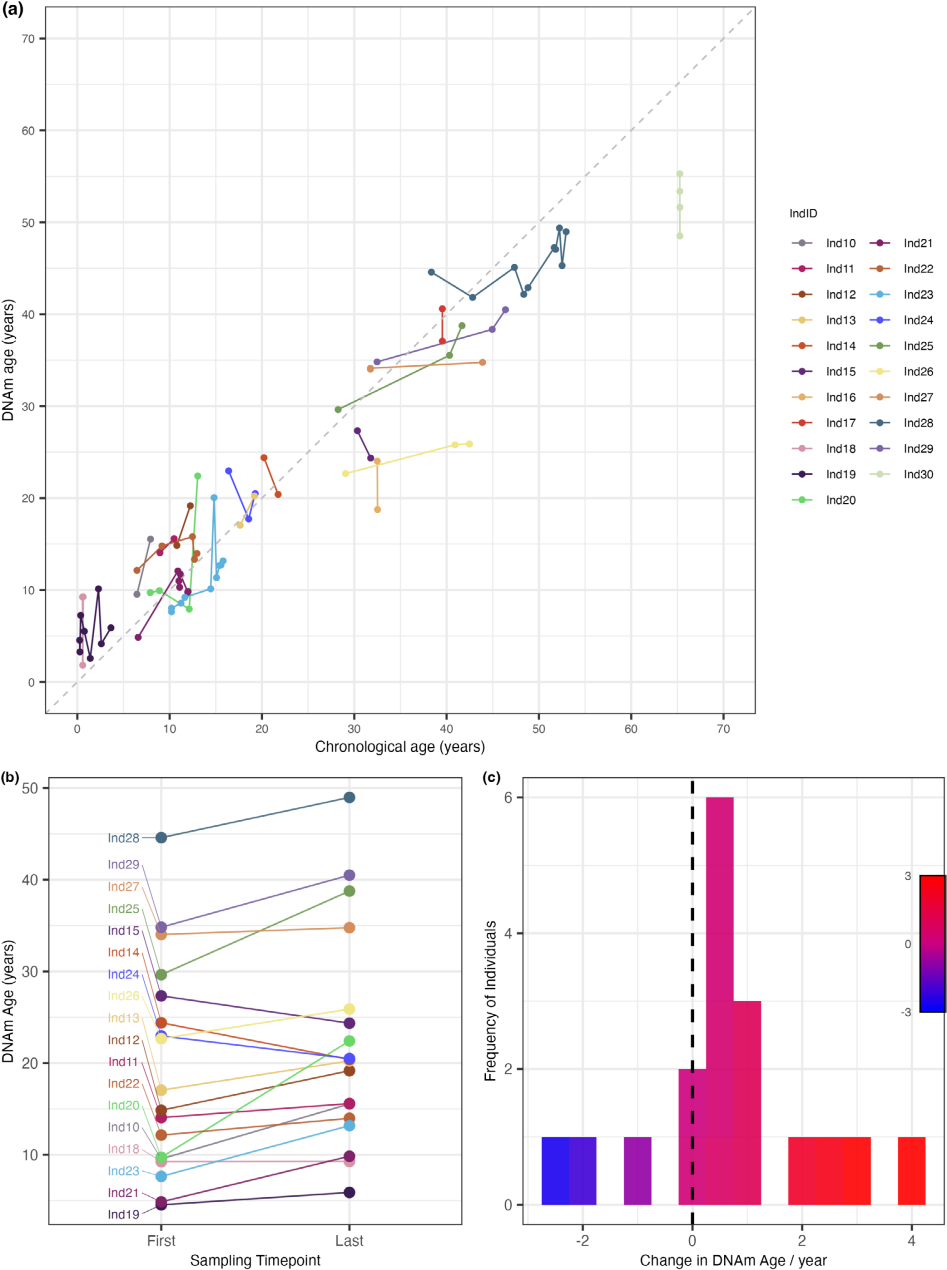
Longitudinal changes in predicted DNA methylation age (DNAm age) across multiple samples from individual Asian elephants. (a) Longitudinal DNAm age trajectories for 21 individuals sampled at multiple time points. Each coloured line represents a single individual (IndID), with points indicating individual sampling occasions plotted against chronological age. (b) Paired comparisons of DNAm age at first and last sampling time points for each individual. Lines connect repeated samples from the same individual and are colour‐coded by IndID to facilitate tracking of individual trajectories over time. Individuals included in this panel were filtered to have a time interval between first and last sampling ranging from 1 to 14 years (*n* = 17). (c) Distribution of the change in DNAm between first and last timepoints across filtered individuals. The vertical dashed line indicates zero change in DNAm age. Bar colours reflect the magnitude and direction of change, with blue indicating decreases and red indicating increases in DNAm age.

Longitudinal analysis using 21 individuals sampled at multiple timepoints (mean interval = 5.02 years, range = 0–14.60 years) revealed variations in methylation patterns over time, with an overall tendency of DNAm age increasing across individuals (Figure 2a). When limiting the comparison to first and last time points per individual (*n* = 18, mean interval = 6.26 years, range = 1.47–14.60 years), 14 individuals showed a general increase in DNAm age over time (*t*‐test: *t* = 1.70, df = 17, *p* = 0.05; Figure 2b,c). Although three individuals showed a decrease in predicted epigenetic age, the overall trend across individuals was positive, supporting the ability of the clock to capture age‐associated change over time.

### 3.4 Age Group DNA Methylation Differences and Performance

**FIGURE 3:**
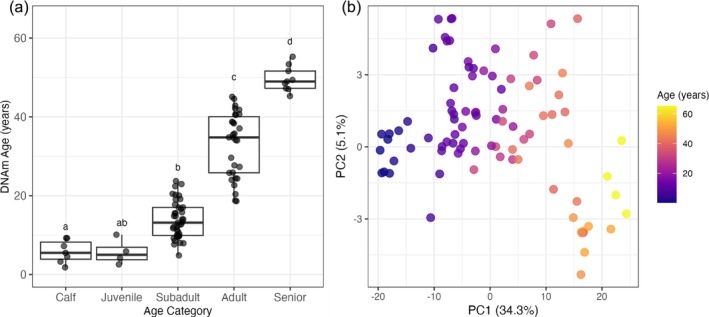
Differences in DNA methylation age (DNAm age) across life stages and methylation structure. (a) Boxplot of DNAm age estimates across five predefined age categories: calves (< 1 year; *n* = 7), juvenile (1–5 years; *n* = 4), subadults (5–20 years; *n* = 41), adults (20–50 years; *n* = 30) and seniors (> 50 years; *n* = 9). Boxplot shows the median and interquartile range, with points representing all samples. Different letters indicate statistically significant differences between groups based on post hoc pairwise comparisons following a one‐way ANOVA (*p* < 0.05). (b) Principal component analysis (PCA) of DNA methylation profiles at the 389 CpG sites used in the final epigenetic clock model. Each point represents a sample, coloured by chronological age (blue = younger, yellow/red = older). The dataset comprises 91 blood samples collected from 30 individuals, including 21 longitudinally sampled individuals (Data S1).

DNAm age differed significantly among the five predefined age categories (one‐way ANOVA, *F* = 40.25, *p* = 2e‐16; Figure 3). DNAm age estimates increased across life stages, with group means as follows: calves (mean = 5.84 ± 1.09 years; range = 1.83–9.26), juveniles (mean = 5.69 ± 1.63 years; range = 2.57–10.13), subadults (mean = 13.83 ± 0.73 years; range = 4.86–23.73), adults (mean = 32.99 ± 1.49 years; range = 18.62–45.10) and seniors (mean = 49.64 ± 1.07 years; range = 45.29–55.28). However, pairwise separation was limited between younger adjacent groups, particularly between calves and juveniles (*p* = 0.99), and between juveniles and subadults (*p* = 0.69) (Figure 3a; Table S2). Additionally, PCA further confirmed the capacity of the 389 CpG sites to distinguish samples by age. Individuals were separated along PC1, which was strongly associated with age (Figure 3b), supporting the clock's ability to capture age‐dependent methylation variation. The PCA trajectory also suggested nonlinear trends in methylation patterns with age, especially as age advances (Figure 3b).

### 3.5 Sex Associated DNA Methylation

Of the 389 CpG sites, only two exhibited significant sex differences, with males driving the association (Figure S2). However, Δage residuals did not differ significantly between sexes (*p* = 0.56; Figure S3a), and this result remained unchanged when controlling for chronological age (*p* = 0.81; Figure S3b). Additionally, no clear sex‐based clustering was observed in the PCA space, further indicating that sex has minimal influence on age‐associated DNA methylation patterns for this epigenetic clock model (Figure S3c). However, sex‐based analyses were exploratory and underpowered due to the limited number of male individuals (*n* = 3), and therefore, the absence of detected sex differences should not be interpreted as evidence that sex effects are absent.

### 3.6 Characteristics of Age‐Related CpGs and Associated Genes

**FIGURE 4:**
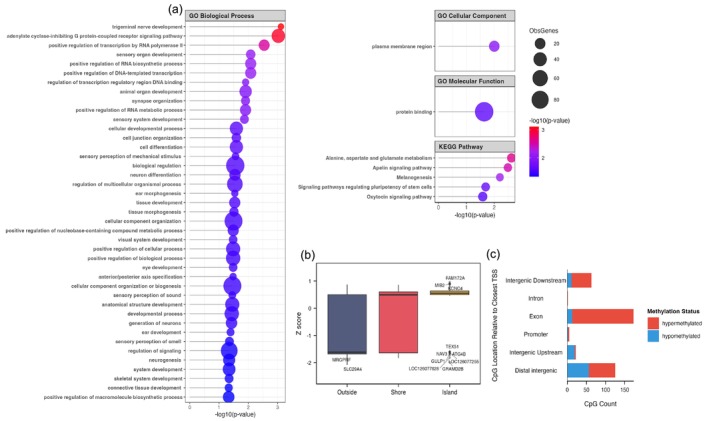
Functional enrichment and genomic distribution of age‐related CpG sites in Asian elephants. (a) Gene ontology (GO) and KEGG pathway enrichment analysis of genes associated with significant age‐related CpG sites in Asian elephants, identified using Enrichr and g:Profiler, with the human genome (hg38/GRCh38) as the reference background. (b) Boxplot showing DNA methylation ageing patterns by CpG density: outside, shore and island regions. (c) Genomic distribution and methylation status of age‐related CpG sites relative to the closest transcriptional start site (TSS).

Out of 148 genes that corresponded with the 389 CpG sites, 129 genes (89%) were successfully mapped for enrichment analysis. In addition to examining potential age‐associated genes, deeper insights can be gained by understanding the pathways, clinical outcomes and regulatory networks linked to these elements. Such enrichment analysis identified biological processes related to development, transcriptional regulation and cellular differentiation, including tissue development, nervous system development and positive regulation of transcription that are predicted to be affected by age‐related CpGs (Figure 4a; Data S2). Notably, two genes: *TIFAB* and *POU4F1* were linked to trigeminal nerve development, which plays a role in somatosensory signalling. In terms of molecular function, age‐associated genes were significantly enriched for protein binding, while cellular component terms highlighted enrichment in the plasma membrane region (Figure 4a). KEGG pathway analysis revealed significant enrichment in signalling pathways involved in melanogenesis, oxytocin signalling and the apelin signalling pathway (Figure 4a).

Age‐related CpGs were predominantly located within CpG islands and exhibited the strongest age‐related hypermethylation, while those in shores and outside regions showed weaker or opposite trends (Figure 4b; Data S2). Methylation levels increased progressively from outside to shore to island regions (ANOVA: *F* = 116.5, *p* = 2e‐16), consistent with known ageing signatures involving hypermethylation in CpG islands and hypomethylation in other genomic regions. Most age‐related CpGs were located in exons and showed hypermethylation trends (Figure 4c). For instance, CpG sites in exon regions of *FAM127A*, *KCNC4* and *MIB2*, all within CpG islands, showed strong age‐related hypermethylation. In contrast, hypomethylation was observed in CpG islands downstream of *GRAMD2B* and *NAV3*, and in regions located outside of CpG islands, upstream or downstream of *SLC29A4* and *MRGPR*, respectively (Figure 4b; Data S2).

## 4 Discussion

### 4.1 Genome‐Wide Epigenetic Clock Model for Asian Elephants

The development of a highly accurate epigenetic clock for Asian elephants contributes primarily to the comparative biology of ageing by providing genome‐wide insight into age‐associated DNA methylation patterns in a long‐lived mammal. Using RRBS and machine‐learning approaches, we identified 389 CpG sites strongly correlated with chronological age from an initial set of 144,611 candidate sites, which were subsequently integrated using elastic net regression to build a robust epigenetic clock. The final model showed high predictive performance (*r* = 0.96 and MAE = 4.82 years), corresponding to a relative error of 6.06% based on the maximum reported lifespan of Asian elephants. This level of performance is comparable to that reported in humans and other non‐model species (relative error: 4.2%–8.5%; Horvath 2013; Stubbs et al. 2017), supporting the broader applicability of epigenetic clocks across diverse mammalian life histories. Although the accuracy of this blood‐based epigenetic clock exceeds that of conventional age estimation methods used in Asian elephants (e.g., morphometrics or tooth wear) (Arivazhagan and Sukumar 2008; Fernando et al. 2022; Mohanarangan et al. 2022), its practical application is constrained by the invasive and logistically demanding nature of blood sampling, particularly in wild populations. Accordingly, the present approach should be viewed as a complementary tool that advances mechanistic understanding of ageing and provides a benchmark for evaluating alternative, less invasive epigenetic methods, rather than as a universally deployable method for conservation monitoring.

To date, only two DNA methylation‐based models have been developed for Asian elephants. Prado et al. (2021) constructed a model using the MammalMethylChip40 array, which targets approximately 36 k conserved CpG sites, and reported a MAE of 3.41 years. In contrast, a methylation‐sensitive high‐resolution melting (MS‐HRM) approach targeting two candidate gene regions (*TET2* and *RALYL*) achieved a MAE of 7.36 years (Arai et al. 2023). Although the array‐based model reported a lower MAE than the present study, the magnitude of this difference should be interpreted cautiously, as prediction accuracy is sensitive to sample composition, age distribution and cohort structure. Thus, variation in these factors between studies may partly explain discrepancies in performance, rather than reflecting inherent methodological superiority. Importantly, these approaches are optimised for different objectives. The array‐based model relies on a pre‐designed set of conserved CpG probes optimised for cross‐species applications (Horvath and Raj 2018), while the RRBS‐based approach enables de novo identification of age‐related CpGs directly from the Asian elephant genome. This allows a more flexible and species‐specific exploration of epigenetic ageing patterns at genome‐wide resolution, including regions not captured by conserved probe sets. Consistent with this, our analysis identified novel age‐associated regions, including *FAM172A*, *MIB2*, *MRGPRF* and *SLC29A4*, alongside shared regions such as *KCNC4*, while other loci reported by Prado et al. (2021), including *ZFHX3*, were not detected in our dataset. Although MS‐HRM offers a cost‐effective and accessible approach, it is inherently limited by its small number of target sites and lower resolution, restricting its ability to capture complex methylation patterns. Notably, while CpG sites in the MS‐HRM target regions were not detected in our dataset, we identified *ELOVL2* (Data S2), a well‐established ageing marker that showed no association with age in the MS‐HRM study (Arai et al. 2023). These comparisons highlight that RRBS provides a balance between cost‐efficiency and genomic coverage, making it well‐suited for non‐model organisms with limited prior methylation data (Balard et al. 2024). However, RRBS does not capture the entire genome and may miss age‐informative CpGs in low‐density or repetitive regions (Arneson et al. 2022; Balard et al. 2024). Future studies could address this limitation using WGBS or long‐read sequencing technologies, which offer more comprehensive genomic coverage and may reveal additional conserved and functionally relevant ageing markers (Lin et al. 2023; Balard et al. 2024; Sigurpalsdottir et al. 2024). Nevertheless, the high cost and complexity of WGBS and long‐read sequencing techniques currently limit their feasibility for large‐scale or routine applications. Despite these limitations, the high accuracy of the RRBS‐based model demonstrates that this approach is sufficient for developing a reliable epigenetic clock in Asian elephants, while also providing valuable insight into the molecular architecture of ageing.

Although the sample size was moderate and multiple samples were obtained from some individuals, introducing potential pseudo‐replication, the use of LOIOCV mitigated this issue and demonstrated robust model generalisability. While larger datasets will be important for further improving model performance and extending its applicability (Anastasiadi and Piferrer 2023), previous studies have shown that accurate epigenetic clocks can be developed from relatively small sample sizes when appropriate validation strategies are employed (Bors et al. 2021; Shealy et al. 2025; Wright et al. 2018). One study even suggests that epigenetic clock performance may be largely independent of sample size under certain conditions (Tangili et al. 2023). Importantly, the inclusion of longitudinal samples from the same individuals, although analytically challenging, provides valuable insight into within‐individual ageing trajectories in long‐lived species. When appropriately accounted for, such data represent a key strength rather than a limitation.

### 4.2 Biological and Methodological Sources of Variation in Epigenetic Age

Longitudinal analyses revealed heterogeneous epigenetic ageing trajectories among individuals. While most individuals showed increases in DNAm age over time, consistent with chronological ageing, others exhibited relatively stable trajectories, and a small subset showed apparent age deceleration. Survival bias may contribute to these patterns, as individuals that reach advanced ages in captivity may represent a healthier subset with slower epigenetic ageing, potentially leading to reduced or neutral age acceleration at older ages. These results should, however, be interpreted with caution, given the model error (MAE = 4.82 years) and the wide range of sampling intervals between repeated measures (1–14 years). In particular, short intervals (< 1–2 years) may fall within the prediction error and therefore be insufficient to detect biologically meaningful changes in DNAm age in a long‐lived species such as elephants. Similarly, studies in humans suggest that epigenetic age changes over short timeframes (within 2 years) are typically modest and context‐dependent (Hamaya et al. 2025), although intra‐annual variation has been reported in other mammals, including felids (Qi et al. 2024). Although this suggests that caution is warranted when interpreting short‐term longitudinal changes in Asian elephants, one individual (Ind30) in our dataset showed a notable increase in DNAm age over a brief interval that coincided with documented health deterioration preceding death. While this observation suggests a potential association between health decline and accelerated epigenetic ageing, the limited sample size and proximity of sampling points relative to model error preclude formal statistical inference. It is known that several potential confounding factors may influence epigenetic ageing trajectories, such as health status, pregnancy, infectious disease exposure, and environmental conditions (Oblak et al. 2021). However, health records and reproductive status were not uniformly available across individuals or time points, preventing systematic evaluation of these factors and representing an important limitation of the present study. Although such studies remain limited in non‐model species, studies on the social status of naked mole rats ( *Heterocephalus glaber* ) and baboons (*Papio*) (Anderson et al. 2021; Kerepesi et al. 2022), as well as hibernation in ground squirrels ( *Ictidomys tridecemlineatus* ) and yellow‐bellied marmots (*Marmota flaviventer*) (Alvarado et al. 2015; Pinho et al. 2022), have demonstrated similar phenomena of epigenetic age acceleration or deceleration, whereby epigenetic age can temporarily speed up or slow down. Given their long lifespans and complex social systems, elephants represent a promising system for investigating the biological drivers of epigenetic ageing. To date, such effects have not been systematically examined in this species. Future studies integrating longitudinal methylation data with detailed health, reproductive and environmental records, particularly in relation to diseases such as elephant endotheliotropic herpesvirus (EEHV) and tuberculosis, may provide important insights into the mechanisms shaping ageing trajectories.

Our model showed variable resolution across life stages, with lower discrimination among younger age groups and reduced precision in senior individuals (Figure 3; Table S3). This pattern likely reflects both biological and methodological factors. Biologically, DNA methylation changes occur most rapidly during early development and puberty, followed by a deceleration or plateau later in life, resulting in non‐linear ageing trajectories rather than strictly linear change (Jylhävä et al. 2017; Shen et al. 2024). This non‐linearity may explain the limited epigenetic separation between adjacent early life stages (e.g., calves and juveniles), as well as reduced resolution at older ages. The lack of clear differentiation among calves, juveniles and subadults may therefore reflect overlapping developmental processes rather than methodological limitations, consistent with evidence from humans showing accelerated epigenetic changes around puberty and adolescence (Shen et al. 2024). Increasing sampling density around key developmental transitions and at older ages would likely improve resolution and clarify age‐specific methylation dynamics. Methodologically, model performance is also influenced by uneven representation across age groups and uncertainty in chronological age estimates for some individuals (Tangili et al. 2023; Newediuk et al. 2025). Expanding sample coverage at both ends of the lifespan may therefore further improve model accuracy and resolution. In practice, integrating this epigenetic approach—which performs well in adults—with traditional morphometric methods that are more informative for younger individuals (Arivazhagan and Sukumar 2008; Fernando et al. 2022), could provide a more holistic framework for demographic assessment. The clear age‐related gradient observed in PCA space further supports the biological relevance of the selected CpG sites and suggests that epigenetic ageing in elephants follows a non‐linear trajectory shaped by developmental and senescent phases, consistent with evolutionary theories of ageing (Bork et al. 2010; Wu et al. 2024). Given their long lifespans, extended post‐reproductive period and minimal reproductive senescence, elephants represent a particularly informative system for exploring how large, long‐lived mammals maintain physiological resilience across the lifespan.

Although no clear sex‐based differences in DNAm age or PCA clustering were detected, this result should be interpreted with caution, given the very limited number of male individuals (*n* = 3) and their restricted age range relative to females. Because male samples were concentrated in younger age classes, any apparent sex differences may be confounded by age‐related effects rather than reflecting true biological variation. While two CpG sites showed subtle male‐biased methylation patterns, the absence of a broader sex effect likely reflects limited statistical power and uneven age representation, rather than true biological equivalence. Accordingly, these findings do not exclude the possibility of meaningful sex‐specific differences in age‐associated DNA methylation. Sex differences in lifespan, mortality and ageing trajectories have been documented in both captive and wild elephant populations, with males typically exhibiting shorter lifespans than females (Lahdenperä et al. 2018; Lemaître et al. 2020). These life‐history patterns suggest that sex‐specific epigenetic ageing dynamics may exist but could not be robustly evaluated here. Future studies incorporating larger and more balanced datasets across sexes and age ranges will be essential to disentangle sex‐specific effects from age‐related variation. Targeted analyses of sex‐associated CpG sites may further improve detection of such patterns, as demonstrated in other species (Peters et al. 2022; Shealy et al. 2025).

### 4.3 Functional Enrichment and Evolutionary Implications

Functional enrichment analysis revealed significant enrichment of age‐related CpG sites in biological processes associated with development, neurogenesis and transcriptional regulation, consistent with findings from other epigenetic ageing studies (Lu et al. 2023). Enrichment of developmental pathways aligns with the theory that ageing could be involved in the reactivation or dysregulation of developmental programmes, potentially reflecting shifts in gene regulation that influence tissue maintenance and repair in adulthood (Wang et al. 2022). Nervous system‐related processes were also enriched, including genes like *TIFAB* and *POU4F1*, which are involved in trigeminal nerve development. This finding is particularly relevant in elephants, given their advanced cognitive abilities and reliance on complex sensory processing (Chusyd et al. 2021). Additional enriched pathways included melanogenesis, oxytocin signalling and apelin signalling, linking age‐related methylation changes to metabolic functions and social or reproductive behaviour. Although melanogenesis is primarily associated with pigment production, it also contributes to oxidative stress regulation and immune function, which are processes known to change with age across mammals (Slominski et al. 2004). Oxytocin signalling plays key roles in social bonding, maternal behaviour and stress regulation (Churchland and Winkielman 2013), all of which are central to elephant social systems. Apelin signalling, which declines with age in humans, is involved in cardiovascular function and energy metabolism and has been linked to age‐related disorders (Chandrasekaran et al. 2008). Although the specific functional roles of these pathways in elephant ageing remain unclear, their enrichment suggests that epigenetic ageing in elephants may influence not only cellular maintenance but also broader physiological and behavioural systems underlying survival, reproduction and social organisation. These findings further indicate that age‐associated methylation patterns may reflect life‐history strategies and ecological adaptations, although additional research is needed to clarify these relationships. The ability of a relatively small subset of CpG sites to predict age with high accuracy suggests a degree of evolutionary conservation in the molecular architecture of ageing. Across the 389 age‐associated CpGs, 288 sites were positively and 101 negatively correlated with age, suggesting that methylation patterns across the genome undergo coordinated regulatory changes of hyper‐ and hypomethylation that likely reflect fundamental ageing processes (Bell et al. 2019). CpG sites showing particularly strong correlations, such as those within *FAM172A* and *SLC29A4* (*r* = ±0.74, *p* = 2.2e‐16), may represent key regulatory loci underlying age‐associated epigenetic change. These sites also provide promising targets for developing more cost‐effective and minimally invasive age estimation approaches in future studies.

Age‐associated DNA methylation in Asian elephants appeared to follow two broad genomic patterns: programmed hypermethylation in functionally important genomic regions and hypomethylation in less critical regions, suggesting a structured and coordinated epigenetic architecture of ageing (López‐Otín et al. 2013; Ciccarone et al. 2018). Hypermethylation was enriched in CpG islands and gene bodies of genes such as *FAM127A*, *KCNC4* and *MIB2*, whereas hypomethylation predominantly occurred in intergenic regions and CpG‐poor regions, including shores and open sea regions, such as *SLC29A4*. These patterns were consistent with a previous finding using microarrays (Prado et al. 2021) and in humans, where age‐related hypermethylation tends to accumulate in regulatory regions, while hypomethylation is more common in CpG‐poor genomic contexts (Wang et al. 2022). Although gene expression was not assessed in this study, such distributions are often interpreted as reflecting differential regulation and stability of methylation across the genome during ageing. For example, CpG‐dense regions are thought to be more tightly regulated, whereas CpG‐poor regions may be more susceptible to stochastic methylation changes over time, potentially reflecting a gradual decline in genomic maintenance mechanisms (Wang et al. 2022). Similar patterns have been observed in other long‐lived mammals (Christensen et al. 2009; Wilkinson et al. 2021), suggesting that fundamental features of epigenetic ageing could be evolutionarily conserved (Lu et al. 2023). The balance between increased regulatory control in functionally important regions and reduced maintenance in less constrained regions may collectively shape the methylation landscape of ageing in Asian elephants and contribute to their extended lifespan. However, functional consequences of these changes cannot be directly inferred and will require integration with transcriptomic data in future studies.

The presence of predictable age‐related methylation changes across diverse mammalian lineages supports DNA methylation as a conserved biomarker shaped by life‐history evolution (Crofts et al. 2024). From an evolutionary perspective, elephants challenge traditional ageing paradigms by lacking a defined post‐reproductive period, maintaining female fertility into old age, and exhibiting prolonged cognitive stability. Our findings suggest that epigenetic clocks capture more than chronological time, reflecting regulatory pathways linked to life‐history traits and ageing strategies. This supports the view that ageing is not a uniformly degenerative process, but one shaped by adaptive regulatory changes across the lifespan (López‐Otín et al. 2013). Understanding how these traits are epigenetically maintained provides insight into comparative ageing biology, particularly in large, long‐lived mammals with slow life histories. As DNA methylation responds to both intrinsic and environmental influences, it may help explain how such traits are maintained across individuals and ecological contexts, offering a valuable framework for future studies.

## 5 Conclusions

The development of an RRBS‐based epigenetic clock for Asian elephants provides a robust molecular framework for investigating age‐associated DNA methylation patterns in a long‐lived, non‐model species. By leveraging genome‐wide methylation profiling, this study identified 389 age‐associated CpG sites and achieved an MAE of 4.82 years, with enrichment in genes and pathways related to neurodevelopment, metabolism, regulatory processes and social behaviour. These findings suggest that epigenetic ageing in elephants reflects not only molecular maintenance, but also broader life‐history traits associated with longevity and social complexity. By identifying species‐specific methylation signatures of ageing, this study contributes to the growing field of comparative ageing biology and offers insight into how epigenetic regulation may be shaped by evolutionary history across mammals. Although reliance on blood samples limits direct application in wild populations, this approach provides a valuable foundation for future studies incorporating alternative sampling strategies, complementary age‐estimation methods, and targeted validation approaches. More broadly, epigenetic clocks offer powerful tools for investigating biological ageing beyond chronological time, with potential applications in studies of health, stress, reproduction and survival, when applied in appropriate ecological and methodological contexts. Expanding epigenetic ageing studies across diverse taxa will be essential for distinguishing conserved and lineage‐specific mechanisms of ageing and for understanding how epigenetic regulation contributes to the evolution of lifespan diversity across the animal kingdom.

## Funding

This work was supported by Japan Science and Technology Agency (JPMJSP2110), Japan Society for the Promotion of Science (25KJ1563, 25H01002, 25K22873) and Environmental Restoration and Conservation Agency by the Ministry of the Environment of Japan (JPMEERF20244M01).

## Ethics Statement

Our research complies with all relevant guidelines and regulations and is reported under the ARRIVE guidelines. All experimental protocols and sample collections were approved by the ethical committee and conducted in strict accordance with the guidelines for the ethics of animal research established by the Wildlife Research Center of Kyoto University (approval number WRC‐2023‐010A and WRC‐2024‐010A). The research and sample collections from Asian elephants were authorised and approved by the management of each participating zoo.

## Conflicts of Interest

The authors declare no conflicts of interest.

## Supporting information


**Figure S1:** (a) Global DNAm level of all 144,611 CpG sites as a function of age (*r* = 0.05, *p* = 0.65). (b) Histogram of Pearson's *r* for relationships between global DNA methylation at each CpG and age across samples.
**Figure S2:** Sex associated differences in DNA methylation levels at individual CpG sites. (a) CpG site located at NC_064822.1_104419887 showed significant sex association in methylation levels (*p* < 0.01), with a stronger association observed in males (*R* ^2^ = 0.77, *p* < 0.0001, *r* = −0.89) compared to females (*R* ^2^ = 0.09, *p* < 0.01, *r* = −0.33). (b) CpG site located at NC_064828.1_107421440 also showed significant sex association (*p* < 0.01), with a stronger association in males (*R* ^2^ = 0.80, *p* < 0.0001, *r* = −0.90) than in females (*R* ^2^ = 0.21, *p* < 0.01, *r* = −0.47).
**Figure S3:** No sex‐associated variation in DNAm age and methylation patterns. (a) Comparison of Δage residuals between females and males (*p* = 0.56) and (b) Δage residuals plotted against chronological age (*p* = 0.81). (c) Principal component analysis (PCA) of DNA methylation profiles from 389 CpG sites used in the epigenetic clock model showed no distinct clustering by sex.
**Table S1:** Model accuracy and precision of all models.
**Table S2:** Results of pairwise t‐tests comparing DNAm age across age group categories.
**Table S3:** The performance of the epigenetic clock at increasing age intervals.

**Data S1:** Sample information.

**Data S2:** Information on CpG sites and enrichment analysis.

## Data Availability

Additional data and R script for this study are available at https://doi.org/10.5061/dryad.ksn02v7kp.
