# Supplementary material for: Epigenetic Signatures of Ageing in Asian Elephants Revealed by Reduced Representation Bisulphite Sequencing
Source: Evol Appl. 2026 Apr 13;19(4):e70236. doi: 10.1111/eva.70236 (PMC13076357; doi:10.1111/eva.70236)
Supplement: Supplementary file 1 — Figure S1: (a) Global DNAm level of all 144,611 CpG sites as a function of age (r = 0.05, p = 0.65). (b) Histogram of Pearson's r for relationships between global DNA methylation at each CpG and age across samples. Figure S2: Sex associated differences in DNA methylation levels at individual CpG sites. (a) CpG site located at NC_064822.1_104419887 showed significant sex association in methylation levels (p < 0.01), with a stronger association observed in males (R 2 = 0.77, p < 0.0001, r = −0.89) compared to females (R 2 = 0.09, p < 0.01, r = −0.33). (b) CpG site located at NC_064828.1_107421440 also showed significant sex association (p < 0.01), with a stronger association in males (R 2 = 0.80, p < 0.0001, r = −0.90) than in females (R 2 = 0.21, p < 0.01, r = −0.47). Figure S3: No sex‐associated variation in DNAm age and methylation patterns. (a) Comparison of Δage residuals between females and males (p = 0.56) and (b) Δage residuals plotted against chronological age (p = 0.81). (c) Principal component analysis (PCA) of DNA methylation profiles from 389 CpG sites used in the epigenetic clock model showed no distinct clustering by sex. Table S1: Model accuracy and precision of all models. Table S2: Results of pairwise t‐tests comparing DNAm age across age group categories. Table S3: The performance of the epigenetic clock at increasing age intervals. [file EVA-19-e70236-s001.docx]

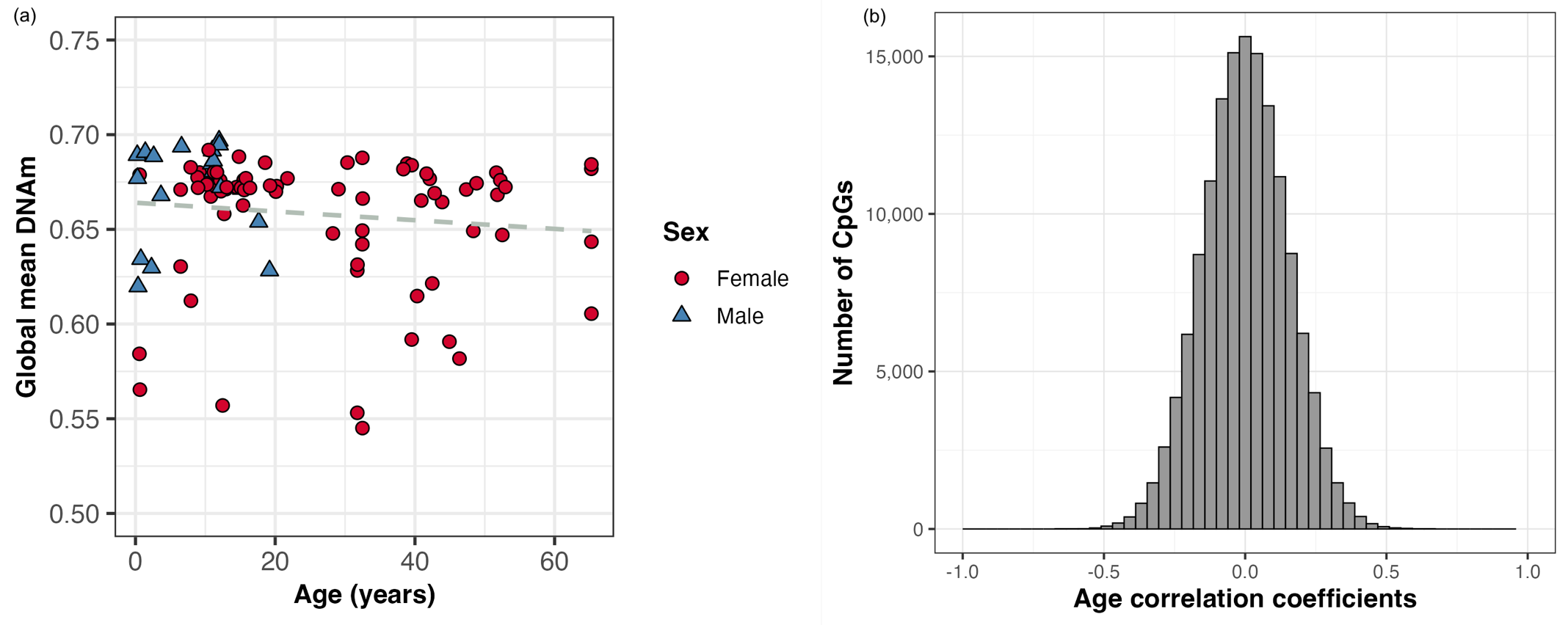


**Supplementary Figure 1.** (a) Global DNAm level of all 144,611 CpG sites as a function of age (*r* = 0.05, *p* = 0.65). (b) Histogram of Pearson’s *r* for relationships between global DNA methylation at each CpG and age across samples.


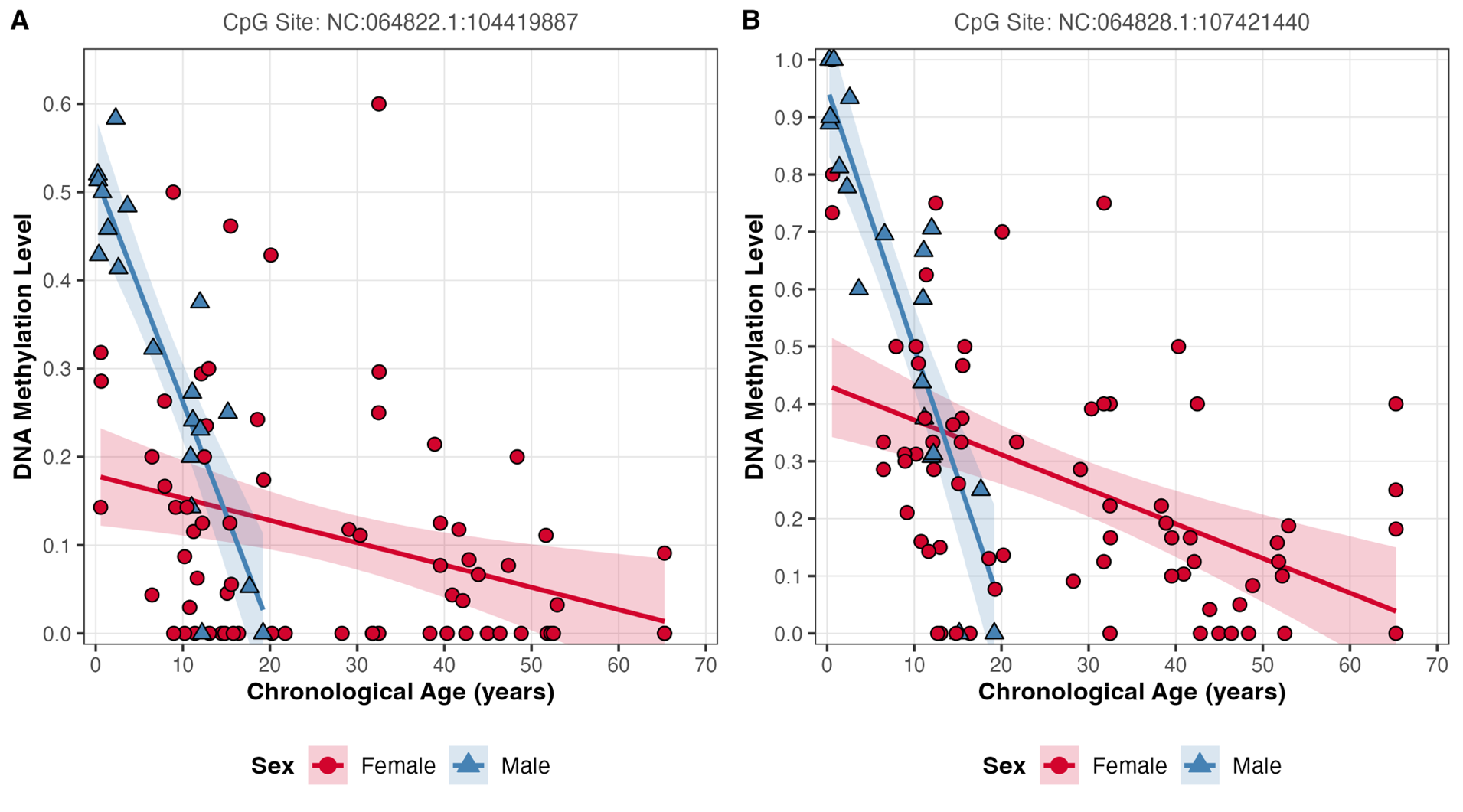


(a)

(b)

**Supplementary Figure 2.** Sex associated differences in DNA methylation levels at individual CpG sites. (a) CpG site located at NC_064822.1_104419887 showed significant sex association in methylation levels (*p* < 0.01), with a stronger association observed in males (*R*^2^ = 0.77, *p* < 0.0001, *r* = –0.89) compared to females (*R*^2^ = 0.09, *p* < 0.01, *r* = –0.33). (b) CpG site located at NC_064828.1_107421440 also showed significant sex association (*p* < 0.01), with a stronger association in males (*R*^2^ = 0.80, *p* < 0.0001, *r* = –0.90) than in females (*R*^2^ = 0.21, *p* < 0.01, *r* = –0.47).


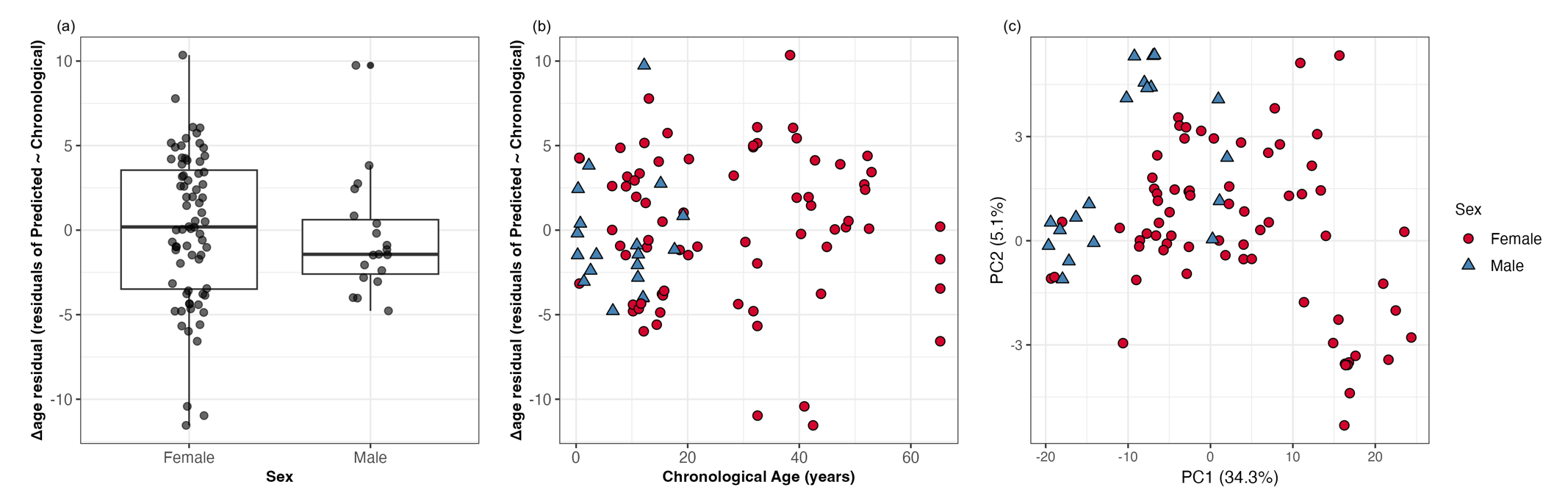


**Supplementary Figure 3.** No sex-associated variation in DNAm age and methylation patterns. (a) Comparison of Δage residuals between females and males (*p* = 0.56) and (b) Δage residuals plotted against chronological age (*p* = 0.81). (c) Principal component analysis (PCA) of DNA methylation profiles from 389 CpG sites used in the epigenetic clock model showed no distinct clustering by sex.

**Supplementary Table 1.** Model accuracy and precision of all models.

| **Feature selection** | | **Regression** | **Train MAE** | **Test MAE** | **MAE difference** | **RMSE** | ***R*^2^** | ***r*** |
| --- | --- | --- | --- | --- | --- | --- | --- | --- |
| **Elastic net** | | | 5.16 | 6.89 | 1.73 | 3.59 | 0.95 | 0.97 |
| **Elastic net** | | **Elastic net** | 1.18 | 6.77 | 5.59 | 4.27 | 0.93 | 0.96 |
| **Elastic net** | | **SVMr** | 2.44 | 6.65 | 4.21 | 4.06 | 0.93 | 0.96 |
| **Cor > 0.5** | | **Elastic net** | 4.47 | 4.82 | 0.35 | 4.21 | 0.91 | 0.96 |
| **Cor > 0.5** | | **SVMr** | 1.74 | 5.04 | 3.30 | 3.60 | 0.95 | 0.97 |
| **Cor > 0.5** | **Elastic net** | **SVMr** | 2.94 | 4.88 | 1.94 | 4.40 | 0.92 | 0.96 |

MAE: mean absolute error, RMSE: root mean square error, *R*^2^: coefficient of determination between DNAm age and chronological age, *r*: Pearson correlation between DNAm age and chronological age (*p-*values were all less than 0.0001). Note that the results of RMSE, *R*^2^, and *r* are from the final age estimation model.

**Supplementary Table 2.** Results of pairwise t-tests comparing DNAm age across age group categories.

|  | **Estimate** | **SE** | **df** | **t.ratio** | ***p*-value** |
| --- | --- | --- | --- | --- | --- |
| **Calf-Juvenile** | 0.156 | 3.67 | 86 | 0.043 | 1.00 |
| **Calf-Subadult** | -7.983 | 2.40 | 86 | -3.331 | 0.011 |
| **Calf-Adult** | -27.149 | 2.46 | 86 | -11.038 | < .0001 |
| **Calf-Senior** | -43.795 | 2.95 | 86 | -14.831 | 0.0700 |
| **Juvenile-Subadult** | -8.140 | 3.07 | 86 | -2.652 | < .0001 |
| **Juvenile-Adult** | -27.305 | 3.12 | 86 | -8.755 | < .0001 |
| **Juvenile-Senior** | -43.951 | 3.52 | 86 | -12.482 | < .0001 |
| **Subadult-Adult** | -19.166 | 1.41 | 86 | -13.614 | < .0001 |
| **Subadult-Senior** | -35.812 | 2.16 | 86 | -16.603 | < .0001 |
| **Adult-Senior** | -16.646 | 2.23 | 86 | -7.475 | < .0001 |

**Supplementary Table 3.** The performance of the epigenetic clock at increasing age intervals.

|  | **Age range** | **Total samples** | **Correlation** | **Mean absolute error** |
| --- | --- | --- | --- | --- |
| **Testing data set** | 5–20 | 18 | 0.65 | 3.04 |
|  | 20–50 | 8 | 0.42 | 8.83 |
| **Training data set** | < 1 | 7 | 0.27 | 5.35 |
|  | 1–5 | 4 | 0.28 | 3.20 |
|  | 5–20 | 23 | 0.61 | 3.58 |
|  | 20–50 | 22 | 0.84 | 3.38 |
|  | > 50 | 9 | 0.76 | 8.39 |
